# Short-Term Complexity of Cardiac Autonomic Control during Sleep: REM as a Potential Risk Factor for Cardiovascular System in Aging

**DOI:** 10.1371/journal.pone.0019002

**Published:** 2011-04-22

**Authors:** Antoine U. Viola, Eleonora Tobaldini, Sarah L. Chellappa, Karina Rabello Casali, Alberto Porta, Nicola Montano

**Affiliations:** 1 Centre for Chronobiology, University of Basel, Basel, Switzerland; 2 Department of Clinical Sciences, Internal Medicine II, L. Sacco Hospital, University of Milan, Milan, Italy; 3 The CAPES Foundation, Ministry of Education of Brasil, Brasilia-DF, Brasil; 4 Institute of Cardiology of Rio Grande do Sul, Porto Alegre, Brazil; 5 Department of Technologies for Health, Galeazzi Orthopedic Institute, University of Milan, Milan, Italy; Central Queensland University, Australia

## Abstract

**Introduction:**

Sleep is a complex phenomenon characterized by important modifications throughout life and by changes of autonomic cardiovascular control. Aging is associated with a reduction of the overall heart rate variability (HRV) and a decrease of complexity of autonomic cardiac regulation. The aim of our study was to evaluate the HRV complexity using two entropy-derived measures, Shannon Entropy (SE) and Corrected Conditional Entropy (CCE), during sleep in young and older subjects.

**Methods:**

A polysomnographic study was performed in 12 healthy young (21.1±0.8 years) and 12 healthy older subjects (64.9±1.9 years). After the sleep scoring, heart period time series were divided into wake (W), Stage 1–2 (S1-2), Stage 3–4 (S3-4) and REM. Two complexity indexes were assessed: SE(3) measuring the complexity of a distribution of 3-beat patterns (SE(3) is higher when all the patterns are identically distributed and it is lower when some patterns are more likely) and CCE_min_ measuring the minimum amount of information that cannot be derived from the knowledge of previous values.

**Results:**

Across the different sleep stages, young subjects had similar RR interval, total variance, SE(3) and CCE_min_. In the older group, SE(3) and CCE_min_ were reduced during REM sleep compared to S1-2, S3-4 and W. Compared to young subjects, during W and sleep the older subjects showed a lower RR interval and reduced total variance as well as a significant reduction of SE(3) and CCE_min_. This decrease of entropy measures was more evident during REM sleep.

**Conclusion:**

Our study indicates that aging is characterized by a reduction of entropy indices of cardiovascular variability during wake/sleep cycle, more evident during REM sleep. We conclude that during aging REM sleep is associated with a simplification of cardiac control mechanisms that could lead to an impaired ability of the cardiovascular system to react to cardiovascular adverse events.

## Introduction

The cardiovascular system has a complex neural control, which is based on the interaction across different subsystems, central and peripheral oscillators, feedback and feedforward reflex mechanisms, humoral, metabolic and local factors [1].

Heart rate variability (HRV) comprises a potent tool that has been widely used in physiological and pathological conditions, as a window over autonomic cardiovascular control [2]. The majority of studies on HRV in physiological and pathological conditions investigate autonomic cardiovascular control by means of classical linear tools, such as time and frequency domains [3]–[6]. However, in order to quantify different aspects of the cardiovascular control mainly related to the organization of different subsystems, in the last years an increasing interest has been focused on the evaluation of the complexity of cardiovascular system, which cannot be adequately assessed by means of the classical linear tools.

For such fine-grained analysis, the use of entropy-derived non-linear indices, such as sample entropy, approximate entropy, corrected conditional entropy and Shannon entropy [7]–[12], has been proposed. Various studies have revealed that both physiological and pathological conditions seem to be characterized by a decrease of HRV complexity. As an illustration, in healthy subjects a gradual increase of sympathetic modulation during graded tilt progressively leads to attenuation in complexity [9]. Most importantly, these indices of complexity of the cardiovascular system can assist in the stratification of risk in patients with an increased risk of sudden cardiac death [10], [13]–[15]. Aging is characterized not only by a reduction of the overall cardiovascular variability [16]–[21] but also by a decrease of complexity of the regulatory action of control mechanisms, including neural cardiovascular control, hormone release regulation and cerebral electroencephalographic activity [22], [23]. This significant age-related loss of HRV complexity indicates that cardiovascular regulation may have a reduced ability to specifically control its different underlying subsystems. As a result, there might be an overall deficiency in integration of control mechanisms, which in turn limits the capabilities of the system to adapt and react to perturbations [22]–[24], thus increasing the risk for major cardiovascular adverse events.

Recently, there has been a growing interest on sleep given the fact that numerous sleep disorders, such as insomnia and sleep disordered breathing, are strongly associated with cardiovascular diseases [25]–[27]. Sleep can be considered a complex phenomenon with important modifications of the cardiovascular autonomic regulation: Non-rapid eye movement sleep (NREM; stages 1, 2 and stages 3,4) is characterized by a predominant parasympathetic drive, while the shift toward rapid eye movement sleep (REM) exhibits increased sympathetic modulation and loss of parasympathetic control [28]–[32].

The application of non-linear dynamics to HRV assessment during sleep revealed a wide spectrum of non-linear characteristics distinctive for each sleep stage. In fact, previous data showed that NREM sleep is characterized by an increase of Sample Entropy [33] and a reduced α_2_ exponent and Lyapunov exponent [34], [35]. Conversely, REM sleep is associated with controversial results ranging from an increase of approximate entropy to a decrease in sample entropy and Kolomogorov entropy [33]–[35].

While aging can be associated with the impairment of several control mechanisms [36], [37], few studies have focused on alterations of the cardiac autonomic regulation during sleep [19], [38]. Brandenberger et al [38] described that older subjects can undergo decreased HRV during each sleep stage, together with loss of parasympathetic and increased of sympathetic modulation, in comparison to young subjects. Recently, Schumann and colleagues [39] studied the age-related modifications of fractal organization in heart rate variability during sleep: a non-linear index derived from the detrended fluctuation analysis technique, α_1_, was found to be age-dependent disregarding wake/sleep cycle, while long-term correlation measured by α_2_ differed in NREM compared to REM sleep but was age-independent [39].

However, to our knowledge no studies have investigated how the short-term complexity of HRV fluctuates with age and across different sleep stages and wakefulness. Thus, the aim of our study was to investigate HRV short-term complexity by means of Shannon entropy (SE) and corrected conditional entropy (CCE) during sleep in young and older subjects, in order to characterize a temporal profile whereby higher cardiovascular risk may occur due to the loss of complexity.

## Methods

### Experimental protocol

Study participants were 12 healthy young volunteers (21.1±0.8 years; 10 men, 2 women; body mass index: 21.8±0.8 Kg.m^−2^) and 12 healthy older subjects (64.9±1.9 years; 10 men, 2 women; body mass index: 24.1±0.5 Kg.m^−2^). All the subjects had no history of cardiovascular disease, sleep disorders, drug abuse and medication intake. Selection of participants was based on questionnaires on subjective sleep quality and sleep–wake habits: participants were enrolled if they had Pittsburgh Sleep Quality Index (PSQI) lower than 5 and the habitual sleep-wake cycle was around 23:00 to 7:00. The protocol was approved by the Hospital Ethics Committee of Strasbourg, France, and all subjects gave their written informed consent to participate. One night of habituation was followed by one experimental night, during which the sleep, cardiac, and respiratory recordings were carried out on the participants. Exclusion criteria were more than 10 periodic leg movements per hour and an apnoea-hypopnea index higher than 5. The segments including periodic breathing have been excluded from the analysis.The experimental protocol has been described in details by Brandenberger et al [38]. Briefly, after one night of habituation sleep, participants underwent complete polysomnographic study. Sleep recordings were made from 23:00 to 07:00 h with a sampling frequency of 256 Hz using an Astro-Med EEG system (Grass Instruments, West Warwick, RI, USA). Four electroencephalograms (EEG) (F3, C3, P3 versus A2, and C4 versus A1), one chin electromyogram, and one electro-oculogram were recorded from 23:00 to 07:00 h. They were visually scored at 30-s intervals using standardized criteria published by Rechtschaffen and Kales [40]. Thoracic and abdominal movements were recorded using a Crystal Trace Piezo Respiration Sensor (Astro-Med EEG system).

### Data analysis

After the QRS complexes were detected on the ECG and the apex of the R wave was located using parabolic interpolation, heart periods were automatically calculated on a beat-to-beat basis as the time between two consecutive R peaks (RR). All QRS detections were carefully checked to avoid erroneous detections or missed beats. Occasional ectopic beats were identified and replaced with interpolated RR interval. The series RR = {RR(i), i = 1,…,N}, where i is the progressive cardiac beat number, were linearly detrended. The series length N ranged from 250 to 300 beats. We carefully avoided non-stationary segments, since stationarity is a prerequisite for entropy analyses [8], [9]. Stationarity was assessed based on a test checking the stability of mean and variance as previously described [41].

Calculation of the RR series was performed during pre-sleep wake (W) and the first two complete REM-nonREM cycles identified from the polysomnographic recordings. Successive analysis was carried out according to the sleep stage classification into Wake (W), Stage 1–2 (S1-2), Stage 3–4 (S3-4) and REM.

### Shannon Entropy (SE)

SE was utilized to assess the complexity of the distribution of the patterns RR_L_ = {RR_L_(i) = (RR(i), RR(i-1), …, RR(i-L+1)), i = 1, …,N-L+1)} where L is the pattern length [8] (see Appendix S1 for mathematical details about the estimation of SE). Therefore, SE is a function of L (i.e. SE = SE(L)). SE(L) is an index describing the complexity of the distribution of RR_L_. Indeed, SE(L) is large if the distribution is flat (all the patterns are identically distributed and the pattern distribution carries the maximum amount of information). On the contrary, SE(L) is small if there is a subset of patterns more likely, while others are missing or infrequent (e.g., in a Gaussian distribution). When SE(L) is calculated with L = 1, SE(1) depends on the complexity of the distribution of the RR series, being maximum when RR is identically distributed and smaller for Gaussian or skewed distributions (see Appendix S1 for further details).

### Corrected conditional entropy (CCE)

We applied the CCE proposed by Porta et al [8] to assess complexity of the RR series (see Appendix S1 for mathematical details about the estimation of CCE). The CCE is based on the definition of the conditional entropy (CE). The CE assesses the amount of information carried by the current RR sample (i.e., RR(i)) when L-1 past samples of RR are known (i.e., RR_L-1_(i-1) = (RR(i-1), …, RR(i-L+1))). CE represents the difficulty in predicting future values of RR based on past values of the same series. It is bounded between 0 and SE(1) quantifying the overall amount of information carried by RR. The CE is 0 when future values of RR are completely predictable given RR past values and it is equal to SE(1) when the knowledge of past values of RR is not helpful to reduce the uncertainty of future RR values. CCE is designed to decrease to 0 only when RR was completely predictable, remained to the maximum value (i.e., SE(1)) when RR was fully unpredictable and showed a minimum when the knowledge of past values was helpful to reduce the uncertainty associated to future RR values. The minimum of CCE, CCE_min_, is taken as a measure of the minimum amount of information carried by the series that cannot be derived from its own past values (i.e. a measure of unpredictability of future values when past samples are given). At difference with approximate and sample entropies, the main advantage of using CCE_min_ is to avoid the arbitrary selection of the number of previous samples (i.e. the pattern length L) helpful to predict future values [8], [10]. The use of CCE_min_ guarantees the automatic, user-independent, selection of the pattern length (i.e. the value of L at CCE_min_, L_min_) that allows the maximum reduction of uncertainty about future values (i.e. maximum predictability) (see Appendix S1 for further details).

### Statistical analysis

A two way Analysis of Variance (two- way ANOVA) for non repeated measurements was used to assess statistical differences between mean values in each sleep stage among young and old subjects. Statistically significant difference was considered when p<0.05. When the sample normality test failed, we used a non parametric test, the Tukey test, to evaluate the statistical significance. A Holm-Sidak test, that is a post-hoc test, was used to assess the significance of mean value changes. Values are reported as mean ± SD.

## Results

Young subjects had similar mean RR interval as well as the total variance across the different sleep stages (Table 1).

**Table 1:** Average RR interval and total variance in young and old subjects during different wake-sleep stages.

YOUNG				
	W	S1-S2	S3-S4	REM
**Mean RR (s)**	1.05±0.15	1.15±0.18	1.18±0.18	1.14±0.17
**Variance (ms^2^)**	10335±8577	7979±4712	9032±7475	7114±7744

OLD				
	W	S1-S2	S3-S4	REM
**Mean RR (s)**	0.94±0.12	0.97±0.11*	0.99±0.24*	0.95±0.13*
**Variance (ms^2^)**	3341±3529*	2702±5443*	1040.5±915*	2409±3217*

All values are expressed as mean ± SD.

W =  wake, S1-S2  =  Sleep stages 1 and 2, S3-S4 =  Sleep stages 3 and 4

*p<0.05, significant changes vs Young; § p<0.05, significant changes in each group vs REM; # p<0.05 significant changes in each group vs S3-S4.

**Figure 1:**
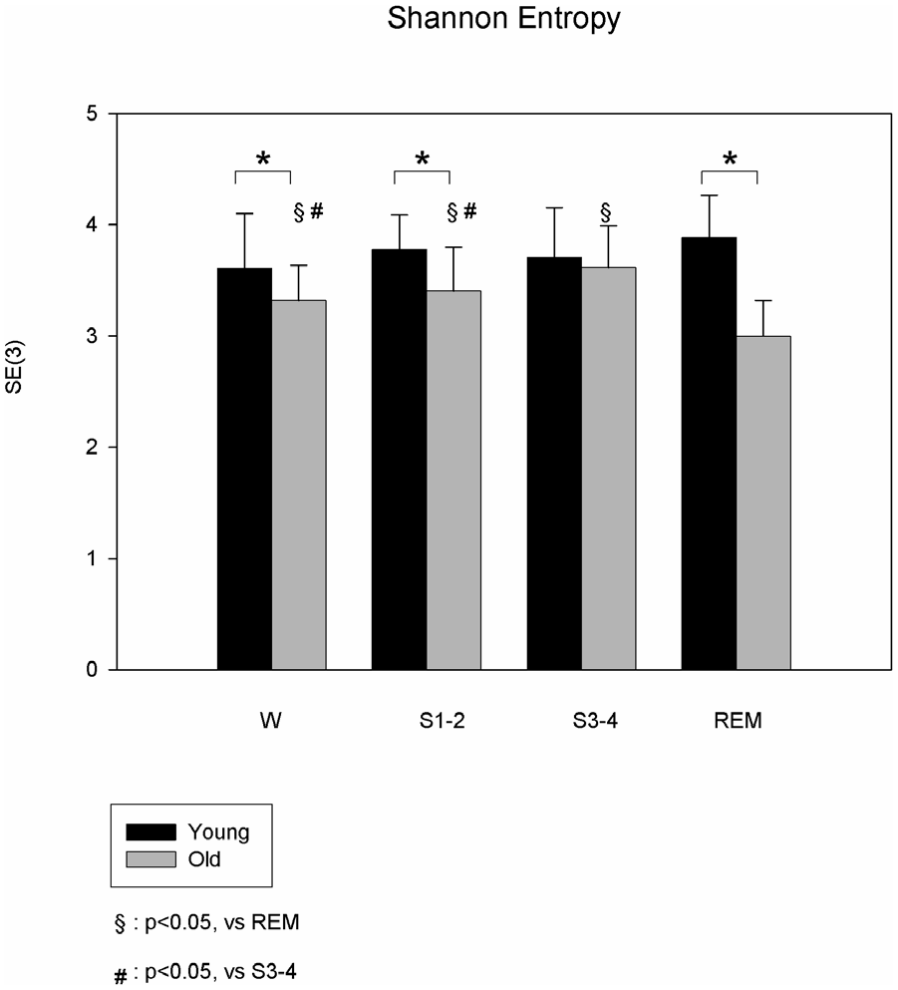
Shannon Entropy during wake and sleep. The graph shows the SE(3) in young (black bars) and in old (grey bars) in wake (W) and during the different sleep stages (S1-1, S3-4 and REM). SE(3) was similar in young subjects between W and the sleep stages. Compared to young, in the old group SE(3) was significantly lower during W, S1-2 and REM. In old subjects, SE(3) was significantly reduced during REM sleep compared to S1-2, S3-4 and W. p<0.05 was considered statistically significant.

**Figure 2:**
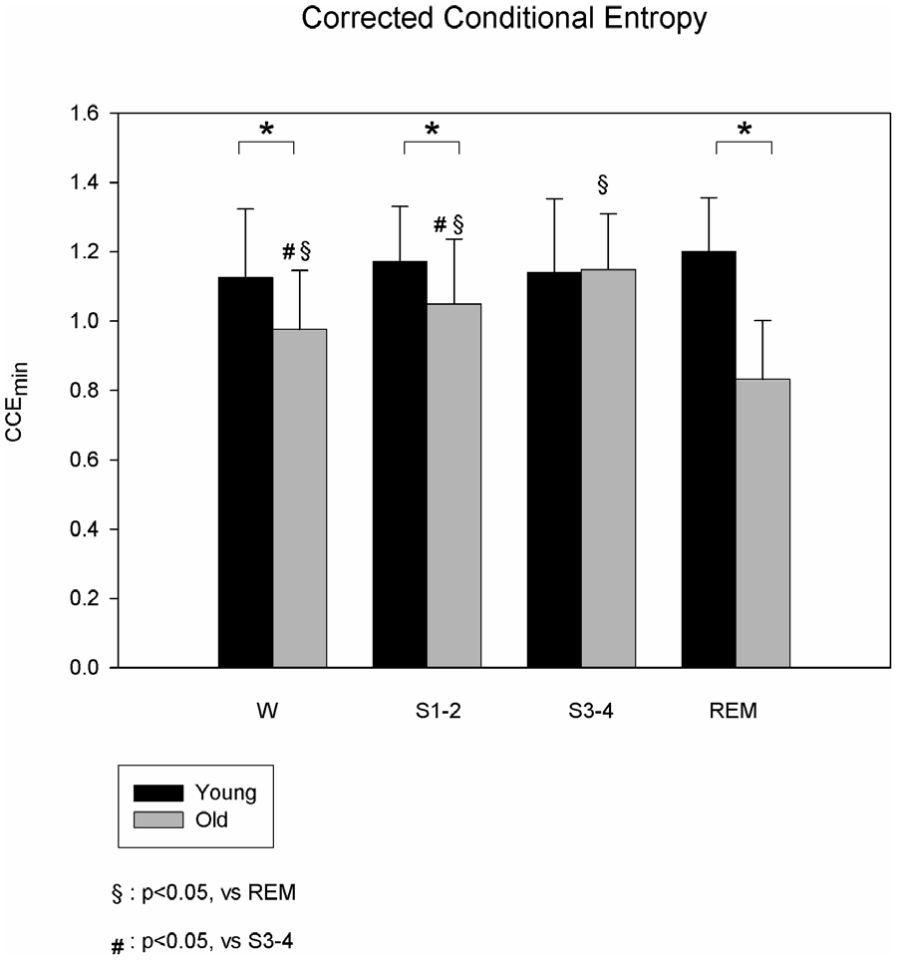
Corrected Conditional Entropy during wake and sleep. The graph shows the CCE_min_ in young (black bars) and old subjects (grey bars) during wake (W) and different sleep stages (S1-2, S3-4, REM). In the young subjects, no differences of CCE_min_ were observed between the sleep stages. Compared to young, the old group showed a significantly lower CCE_min_ in W, S1-2 and REM. In the old participants, CCE_min_ was significantly reduced during REM sleep compared to S1-2, S3-4 and W.

SE(3) and CCE_min_ were not statistically different between the sleep stages (Fig. 1 and 2).

In the older participants, SE(3) and CCE_min_ were significantly reduced during REM sleep compared to S1-2, S3-4 and W (2.99±0.32 vs 3.4±0.39, 3.62±0.38 and 3.32±0.32 respectively for SE (3); 0.83±0.17 vs 1.05±0.19, 1.15±0.16 and 0.98±0.17 respectively for CCE _min_) (Fig. 1 and 2).

The length of the conditioning pattern at CCE_min_, L_min_, was monitored in both populations and in all experimental conditions.

No significant differences were found between old and young subjects and among sleep periods. Therefore, all the values of L_min_ were pooled together. Median of L_min_ was 3 and values ranged from 1 to 6. The median of L_min_ (i.e. 3) was selected as the most representative value of the pattern length able to reduce to the highest level the uncertainty on future RR samples. Thus, SE(L) was sampled at L = 3.

SE(3) and CCE_min_ were not statistically different between the sleep stages.

Comparison of both age groups revealed that the RR interval and the total variance were significantly lower during all the sleep stages in older subjects.

SE(3) was significantly lower during W, S1-2 and REM in older participants, in comparison to young volunteers (3.32±0.32 vs 3.61±0.49, 3.4±0.39 vs 3.77±0.31, 2.99±0.32 vs 3.88±0.38, respectively; p<0.05 for all). Older participants had significant lower CCE_min_, in W, S1-2 and REM (0.98±0.17 vs 1.13±0.12, 1.05±0.19 vs 1.17±0.15, 0.83±0.17 vs 1.20±0.16 vs., respectively, p<0.05 for all) (Fig. 1 and 2). SE and CCE_min_ were not significantly different between young and older during S3-4 (3.62±0.38 vs 3.70±0.44 and 1.15±0.16 vs 1.14±0.21, for SE and CCE_min_ respectively).

## Discussion

Our results indicate that aging can be characterized by a reduction of entropy indices of cardiovascular variability during wake/sleep cycle and that this fall occurs particularly during REM sleep compared to wake and NREM sleep. This suggests a potential reduction in the capability of the cardiovascular system to respond to perturbations and to adapt to stressors during this sleep stage, which in turn might lead to a possible increase in cardiovascular risk. However, while in healthy older participants this phenomenon maybe devoid of clinical relevance, it may turn into an adjunctive state of increased cardiovascular risk when associated to major cardiovascular diseases, such as heart failure and coronary artery diseases. Indeed, these pathological conditions are *per se* associated with impaired cardiovascular variability and reduced complexity of cardiovascular control, which by itself provides an independent negative prognostic risk factor for cardiovascular mortality [13]–[15]. In this context, REM sleep might provide an additional stress load on a cardiovascular system that is already characterized by a reduced capability to respond to perturbations.

In the last decades an increasing interest has been focused on the relationship between autonomic cardiovascular regulation and sleep. The interest in studying this relationship was motivated by the evidence that a strong correlation exists between sleep disorders, such as obstructive sleep apnea (OSA) or chronic sleep deprivation, and increased incidence of cardiovascular morbidity and mortality [26]. One of the major pathophysiological issue in explaining this phenomenon is the important alteration of autonomic cardiovascular control observed in sleep disorders. For example, it has been demonstrated that OSA patients, that are at increased risk for cardiovascular events, have a cardiovascular control characterized by sympathetic overactivity compared to control subjects, both during night-time and day-time [26]. Similarly, aging can be associated to a reduction of the total variance of HRV and a relative predominance of sympathetic modulation of HRV [16]–[21]. The combination of these two aspects – aging and sleep – may thus provide a physiological scenario associated to deregulated autonomic cardiac control, which in turn can result in adverse effects on cardiovascular morbidity and mortality.

However, the complex interaction of the autonomic nervous system, sleep and aging was previously assessed in terms of classical tools of HRV, such as linear spectral approach, with no inferences on the complexity of cardiovascular regulation.

The application of non-linear analysis approaches, such as entropy measures, in several cardiovascular diseases, for instance congestive heart failure, revealed that these patients had a significant loss of the circadian rhythm of HRV dynamics [10], as well as a reduction of entropy measures [13] and an altered fractal organization [14]; in addition, non-linear methods have been demonstrated to be powerful tool for risk stratification in post-myocardial infarction and hypertrophic cardiomiopathy patients[13]–[15].

In our work, we evaluated two different entropy measures of HRV during sleep, carrying different information: SE(3) is an index capable of describing the complexity of the distribution of patterns of length L = 3 (the SE(3) is high when the distribution of patterns is flat, i.e. the patterns are equally distributed, while SE(3) is small when a subset of patterns is more likely); the CCE_min_ is an index of the minimum amount of information that cannot be derived from the knowledge of previous values [7]–[9] (i.e. an index of the unpredictability of the RR series assigned the number of past values maximizing the prediction of future values).

We selected SE(3) and CCE_min_ as measures of complexity because these indexes can be reliably calculated over short data sequences, while indexes such as correlation dimension and Lyapunov exponents require longer series the length of which might be incompatible with the duration of sleep periods and absence of non-stationarities. In addition, CCE_min_ should be preferred to approximate and sample entropies: indeed, approximate entropy provides a biased estimate of complexity [9] and to sample entropy necessitates an arbitrary selection of the pattern length L.

Older subjects revealed significant changes of time domain parameters, i.e. an increase of heart rate and a decrease of total variance compared to young subjects. Considering the entropy measures in the two groups, the older exhibited a significant reduction of entropy indices during wake, S1-2 and REM, in comparison to young, which suggests that aging can be associated with a gradual decline of complexity in cardiovascular control during wake and sleep. In turn, this deregulation of cardiovascular system could be associated with an increased susceptibility to cardiac adverse events.

A decrease of entropy measures can be associated to a shift of the symaptho-vagal balance toward sympathetic predominance [9] and more generally, to a simplification of the cardiovascular regulation arranged over a smaller range of temporal scales. In physiological conditions, different control systems, such as central autonomic oscillators, baroreflex and chemoreflex mechanisms, sympatho-sympathetic reflexes, microvascular regulation and neuroendocrine system, interact in order to regulate cardiovascular system. In pathological conditions, the control system is characterized by the predominance of one of these mechanisms, while the others are less active, inhibited or impaired, thus leading to a decrease of HRV complexity. In the long-term, this simplification of the cardiovascular control reduces flexibility of the cardiovascular system and increases its susceptibility to stressors.

It is worth notice that the assessment of SE and CCE during wake and sleep revealed that in the old group the reduction of the entropy measures is much higher during REM sleep than during wake and NREM sleep. Recently, Shumann et al [39] described the aging effects on cardiac dynamics across the sleep stages: the authors observed that an index used to assess the ability of the deceleration of the sinus node, called the deceleration capacity, was reduced in older volunteers and in REM sleep, hypothesizing that this decrease possibly indicates an increased cardiovascular risk with aging during NREM sleep and REM. However, this index does not reflect a change of complexity of cardiovascular control. Conversely, in our study, considering the different sleep stages, the entropy measures were significantly reduced during REM sleep compared to wake and NREM sleep. Of note, we consider as “Wake state”, only the pre-sleep period, because this stage is uneffected by sleep transitions in comparison to wake after sleep onset (WASO) and morning awakening stages [42].

Thus, our results can lead to the hypothesis that during ageing REM, more than NREM sleep, is a condition characterized by a marked reduction in cardiovascular control complexity. As it has been already shown that a decrease in HRV complexity is associated with an increase in cardiovascular risk [13]–[15], our data could suggest that in old subjects REM sleep, with respect to wake and NREM sleep, can represent a period of major cardiovascular risk, associated to a higher risk for cardiovascular adverse events such as sudden cardiac death.

Our study strengthen the hypothesis that, because complexity is a measure of the capability of cardiovascular system to respond to perturbations, it can be considered an index of increased cardiovascular risk in aging and patients with cardiac diseases, as it has been reported by other investigators [43], [44]. Yet, our data indicate that the complexity analysis was able to unmask a further condition of increased potential risk such as REM sleep. This might partly support the hypothesis that the highest occurrence of acute cardiovascular events in early morning hours, possibly during REM sleep [45], might be related to the impact of sympathetic surges at awakening.

Whether this alteration of complexity may impact on cardiovascular morbidity or mortality needs to be further investigated and future studies are needed in order to evaluate the cardiovascular risk during specific sleep stages in healthy and pathological subjects, suggesting potential high risk moments and possible target for prevention of cardiac major events.

## Supporting Information

Appendix S1 Conditional Entropy, Shannon Entropy and Corrected Conditional Entropy
(DOCX)
