# Supplementary material for: Short-Term Complexity of Cardiac Autonomic Control during Sleep: REM as a Potential Risk Factor for Cardiovascular System in Aging
Source: PLoS One. 2011 Apr 22;6(4):e19002. doi: 10.1371/journal.pone.0019002 (PMC3081328; doi:10.1371/journal.pone.0019002)
Supplement: Appendix S1 — Conditional Entropy, Shannon Entropy and Corrected Conditional Entropy (DOCX) [file pone.0019002.s001.docx]

Appendix S1

*Conditional Entropy, Shannon Entropy and Corrected Conditional Entropy*

Given RR={RR(i), i=1,…,N}, where i is the progressive cardiac beat number and N is the series length, RR series is coarse-grained according to an uniform quantization procedure. Under this quantization technique the full range of RR dynamics (i.e. the difference between the maximum and minimum value) is spread over ξ quantization bins and the values inside each bin are substituted with an integer ranging from 0 to ξ-1 coding the specific bin. Therefore, RR becomes a quantized integer series, i.e. RR^ξ^={RR^ξ^(i), i=1,…,N}. Let us construct patterns from the quantized series using the technique of the delayed coordinates as RR_L_^ξ^(i)=(RR^ξ^(i),RR^ξ^(i-1), …, RR^ξ^(i-L+1)). Let us be RR_L_^ξ^={RR_L_^ξ^(i), i=1,…,N-L+1}, the series of the patterns that can be constructed from RR^ξ^. The conditional entropy (CE) of RR is defined as

 (1)

where p(RR^ξ^(i)/RR^ξ^_L-1_(i-1)) is the conditional probability of RR^ξ^(i) given previous L-1 samples (i.e. the pattern RR^ξ^_L-1_(i-1)) and p(RR^ξ^_L_(i)) is the probability of the pattern RR^ξ^_L_(i) formed by the current value, RR^ξ^(i), and by the L-1 past samples, RR^ξ^_L-1_(i-1). The outer sum is extended from 1 to the number of different patterns RR^ξ^_L_(i)’s (i.e. ξ^L^), while the inner sum from 1 to the number of different samples RR^ξ^(i)’s (i.e. ξ). The CE represents the amount of information carried by the most recent sample of RR when L-1 past samples are given. It is bounded between 0 and SE(1) given by

 (2)

where the sum is extended from 1 to the number of different samples RR^ξ^(i)’s (i.e. ξ). SE(1) represents the maximum amount of information carried by the series RR (i.e. the information carried by RR(i) when no previous conditioning samples are given). SE can be assessed over RR^ξ^_L_ as follows

 (3),

where the sum is extended from 1 to the number of different patterns RR^ξ^_L_(i)’s (i.e. ξ^L^), thus evaluating the amount of information carried by the series of patterns, RR^ξ^_L_,

CE equals 0 when p(RR^ξ^(i)/RR^ξ^_L-1_(i-1))=1 for every pattern RR^ξ^_L-1_(i-1). A p(RR^ξ^(i)/RR^ξ^_L-1_(i-1))=1 indicates that RR^ξ^(i) is completely predictable based on the knowledge of RR^ξ^_L-1_(i-1). If this condition occurs for every RR^ξ^_L-1_(i-1), RR is fully predictable given L-1 past values. Unfortunately, as suggested by Porta et al [1] the above mentioned condition is always encountered while increasing the pattern length L. Indeed, p(RR^ξ^(i)/RR^ξ^_L-1_(i-1))=1 is estimated even when the pattern RR^ξ^_L-1_(i-1) is found only once in RR^ξ^_L-1_. In this case the estimated certainty is not reliable because it is based on a unique appearance of RR^ξ^_L-1_(i-1) in RR^ξ^_L-1_. Since the percentage of patterns found only once grows monotonically towards 100% with L, CE always decreases toward 0 with L independently of the type of RR dynamics. This situation must be prevented because it is fully artificial and solely related to the shortness of the data sequence. In order to prevent the artificial decrease of the information carried by RR given L past samples, we define the corrected CE (CCE)

 (4)

where perc(RR^ξ^_L_) represents the fraction of patterns found only once in RR^ξ^_L_ with 0≤perc(RR^ξ^_L_)≤1. This function decreases towards 0 only in case that RR is perfectly predictable given past RR values, it remains constant when past RR values are not helpful to predict future RR, and it exhibits a minimum when past RR values are only partially helpful to predict RR. The minimum of CCE, CCE_min_ represents the minimum amount of information carried by RR given its own past values: the larger this value, the larger the amount of information carried by RR, the smaller the predictability of RR based on its own past values.

**Reference**

1. Porta A, Baselli G, Liberati D, Montano N, Cogliati C et al. (1998) Measuring regularity by means of a corrected conditional entropy in sympathetic outflow. Biological Cybernetics. 78:71-78.
